# Deconvoluting virome-wide antibody epitope reactivity profiles

**DOI:** 10.1016/j.ebiom.2021.103747

**Published:** 2021-12-16

**Authors:** Daniel R. Monaco, Sanjay V. Kottapalli, Florian P. Breitwieser, Danielle E. Anderson, Limin Wijaya, Kevin Tan, Wan Ni Chia, Kai Kammers, Patrizio Caturegli, Kathleen Waugh, Mario Roederer, Michelle Petri, Daniel W. Goldman, Marian Rewers, Lin-Fa Wang, H. Benjamin Larman

**Affiliations:** a Department of Pathology, Division of Immunology, Institute of Cell Engineering, Johns Hopkins School of Medicine, Baltimore, MD, USA; b Center for Computational Biology, McKusick-Nathans Institute of Genetic Medicine, Johns Hopkins University School of Medicine, Baltimore, MD 21205, USA; c Programme in Emerging Infectious Diseases, Duke-NUS Medical School, 8 College Road, 169857, Singapore; d Department of Infectious Diseases, Singapore General Hospital, 20 College Road, 169856, Singapore; e National Neuroscience Institute, 11 Jalan Tan Tock Seng, 308433, Singapore; f Department of Oncology, Division of Biostatistics and Bioinformatics, The Sidney Kimmel Comprehensive Cancer Center at Johns Hopkins, The Johns Hopkins University School of Medicine, Baltimore, MD, USA; g Barbara Davis Center for Diabetes, University of Colorado Denver, Aurora, CO, USA; h ImmunoTechnology Section, Vaccine Research Center, National Institute of Allergy and Infectious Diseases, National Institutes of Health, Bethesda, MD, USA; i Department of Medicine, Division of Rheumatology, The Johns Hopkins University School of Medicine, Baltimore, MD, USA

**Keywords:** Phage ImmunoPrecipitation Sequencing (PhIP-Seq), VirScan, Encephalitis, Type 1 diabetes, Systemic lupus erythematosus, Antibody profiling

## Abstract

**Background:**

Comprehensive characterization of exposures and immune responses to viral infections is critical to a basic understanding of human health and disease. We previously developed the VirScan system, a programmable phage-display technology for profiling antibody binding to a library of peptides designed to span the human virome. Previous VirScan analytical approaches did not carefully account for antibody cross-reactivity among sequences shared by related viruses or for the disproportionate representation of individual viruses in the library.

**Methods:**

Here we present the AntiViral Antibody Response Deconvolution Algorithm (AVARDA), a multi-module software package for analyzing VirScan datasets. AVARDA provides a probabilistic assessment of infection with species-level resolution by considering sequence alignment of all library peptides to each other and to all human viruses. We employed AVARDA to analyze VirScan data from a cohort of encephalitis patients with either known viral infections or undiagnosed etiologies. We further assessed AVARDA's utility in associating viral infection with type 1 diabetes and lupus.

**Findings:**

By comparing acute and convalescent sera, AVARDA successfully confirmed or detected encephalitis-associated responses to human herpesviruses 1, 3, 4, 5, and 6, improving the rate of diagnosing viral encephalitis in this cohort by 44%. AVARDA analyses of VirScan data from the type 1 diabetes and lupus cohorts implicated enterovirus and herpesvirus infections, respectively.

**Interpretation:**

AVARDA, in combination with VirScan and other pan-pathogen serological techniques, is likely to find broad utility in the epidemiology and diagnosis of infectious diseases.

**Funding:**

This work was made possible by support from the National Institutes of Health (NIH), the US Army Research Office, the Singapore Infectious Diseases Initiative (SIDI), the Singapore Ministry of Health's National Medical Research Council (NMRC) and the Singapore National Research Foundation (NRF).

**Research in context**

**Evidence before this study**

Anti-viral antibody profiling has the potential to enable unbiased diagnosis of infectious diseases and to uncover novel epidemiologic associations. VirScan is a programmable bacteriophage display system developed to profile serum antibodies using overlapping 56 amino acid peptides that tile across all human viruses. Interpreting data from VirScan or related assays is difficult, in large part due to signals associated with antibody cross-reactivity. The lack of an approach to deconvolute antibody profiles has limited the utility of VirScan and related technologies in both clinical and research settings.

**Added value of this study**

Here we present a novel analytical framework, the AntiViral Antibody Response Deconvolution Algorithm (AVARDA), which enables deconvolution of VirScan data and provides a probabilistic assessment of species-level antibody responses. AVARDA was established using a set of samples from an encephalitis cohort and then applied to a longitudinal type 1 diabetes cohort, as well as a cross-sectional lupus cohort. AVARDA significantly improved the rate of diagnosing viral encephalitis and identified biologically plausible associations between viral responses and these autoimmune diseases.

**Implications of all the available evidence**

AVARDA empowers highly multiplexed antibody profiling via a statistical treatment of antibody cross-reactivity and epitope redundancy. The algorithm generates useful summary statistics, including p-values of infection and response breadths, which can be used for enhanced diagnosis and unbiased viral epidemiology.

Alt-text: Unlabelled box

## Introduction

Unbiased profiling of antiviral antibody binding specificities has broad utility for epidemiological investigations, surveillance for emerging viruses, and the diagnosis of infections.^1^, ^2^, ^3^, ^4^ Phage ImmunoPrecipitation Sequencing (“PhIP-Seq”)^5^ with a peptide library spanning the human virome (“VirScan”)^6^ provides a platform for comprehensive, high-throughput, low-cost analysis of antiviral antibodies. While other multiplexed serological techniques exist,^7^ each is limited in its representation of viral antigens,^8^ the size and quality of the epitopes presented,^9^ the per-sample assay cost and/or sample throughput. VirScan provides excellent performance characteristics, but interpretation of assay results has been limited by underdeveloped analytical approaches.

Our previously published approach suffers from three critical limitations. The number of unique, non-overlapping, virus-associated antibody specificities (a measure of response "breadth" or “clonality”) conveys important biological information and determines the confidence of a predicted exposure. Previously, non-overlapping specificities were defined using a rudimentary heuristic that typically underestimated response breadth. Second, a significantly reactive peptide was considered only in the context of the specific virus it was designed to represent. This ignored sequence homology between related viruses, and any potential for antibody cross-reactivity. Further, the VirScan library was designed to cover single representative proteins from UniProt clusters of 90% identity. Relying solely upon the intended viral representations of reactive peptides to diagnose infections will therefore result in both false negative results (“missing” proteins from highly similar organisms) and false positive results (reactivity due to unappreciated cross-reactive antibodies). Third, we previously relied on each virus's proteome “size” to establish virus-specific thresholds for seropositivity. This approach ignored the proportional representation of each virus within the reactive set of peptides and the overall representation of each virus in the library. Additionally, using a binary threshold for seropositivity is far less informative than a probabilistic assessment of a viral infection.

Here we introduce the AntiViral Antibody Response Deconvolution Algorithm (AVARDA), a systematic framework for probabilistic analysis of highly multiplexed antiviral antibody epitope reactivity data. AVARDA integrates three key modules to generate a conservative and probabilistic assessment of antibody responses. The first module uses sequence alignment to define each reactive peptide's relationship to a comprehensive database of all human viral genomes, which have been translated in all six reading frames. This permits a conservative elaboration of all peptide reactivities that could be associated with each potential viral infection. The second module constructs a sequence homology-based network graph for each virus's reactive peptides, so as to define the minimum number of independent specificities (response breadth) required to produce the graph. The third module iteratively assigns each peptide to its most likely associated viral infection(s), according to a null model that considers the overall representation of each virus in the VirScan library. We permit individual peptides to be associated with multiple distinct infections, provided there is sufficient evidence for each viral infection on its own (i.e. in the absence of the shared peptides). AVARDA also indicates uncertainty in peptide-virus assignments when there is insufficient evidence to discriminate between infections by related viruses, but when there is sufficient evidence to conclude that an infection has indeed occurred. Linking these modules, the final output of AVARDA provides p-values adjusted for multiple hypothesis testing for exposure to each virus, along with the associated breadths of the antibody responses and the relationships between the reactive peptides.

The utility of antibody-based diagnostics has been constrained by an inability to robustly discriminate among infections by closely related viruses.^10^ Highly multiplexed analyses of antiviral antibodies provides the potential to distinguish among related viruses, but requires a robust consideration of cross-reactivities. Quantifying an individual's change in IgG specificities over time can provide clear evidence for an evolving immune response to a single virus. We have therefore developed and tested the AVARDA algorithm using acute and convalescent VirScan data from patients with viral encephalitis. The predictions of the algorithm were found to agree with clinical nucleic acid or serologic testing in the majority of cases. Utilizing VirScan/AVARDA analysis in conjunction with traditional nucleic acid testing resulted in a 44% increase in the diagnosis of viral encephalitis. Finally, we demonstrate the utility of AVARDA for investigating the association of viral infections with development of autoimmune type 1 diabetes (T1D) and systemic lupus erythematosus (SLE).

## Methods

### Serum samples

#### SNIP study

The Singapore Neurologic Infections Program (SNIP) is a prospective cohort approved by the Singhealth Centralised Instituitional Review Board (CIRB Ref: 2013/374/E). The SNIP study aims to describe the epidemiology of CNS infections in Singapore; improve the diagnosis of etiologies of CNS infections through a systematic clinical, laboratory and neuroradiological evaluation and extensive diagnostic testing; evaluate the prognosis, long-term outcomes and socio-economic costs of CNS infections; and establish an archive of biological tissues from patients with encephalitis and CNS infections that can be utilized for future testing for emerging pathogens or non-infectious etiologies.

Subjects were enrolled from 6 hospitals in Singapore (Singapore General Hospital, Tan Tock Seng Hospital, National University Health System, Changi General Hospital, Khoo Teck Puat Hospital, Kandang Kerbau Hospital). Individuals, both male and female, more than one month of age, were enrolled upon admission to the hospital with clinical suspicion for CNS infections, or one or more of the following: fever or history of fever (≥ 38 °C) during the presenting illness; seizures; focal neurological deficits; CSF white cell count pleocytosis (> 4 WBC/ µL); abnormal neuroimaging suggestive of CNS infection; abnormal electroencephalogram suggestive of CNS infection; depressed or altered level of consciousness, or no alternative etiology for acute paralysis identified. Patients with indwelling ventricular devices such as EVD and ventriculo-peritoneal shunts were excluded from the study. Blood samples were be obtained on enrollment into the study and then at 2 weeks after enrollment or at time of discharge. Informed consent was obtained from all patients.

#### DAISY longitudinal study

The Diabetes Auto Immunity Study in the Young (DAISY) [NCT03205865] is a prospective study of children with increased risk for Type 1 Diabetes. The details of the newborn screening ^11^ and follow up^12^ have been previously published. Per protocol, serum was tested at 9, 15, and 24 months and, if autoantibody negative, annually thereafter; children found to be autoantibody positive were re-tested every 3–6 months. Recruitment took place between 1993 and 2004 and follow-up results are available through February 2018. Written, informed consent was obtained from the parents of study participants. The Colorado Multiple Institutional Review Board approved all study protocols.

#### Cross-sectional lupus and control cohorts

Systemic lupus erythematosis (SLE, lupus) sera came from the Hopkins Lupus Cohort. All patients met SLICC classification criteria for SLE.^13^ The Hopkins Lupus Cohort was approved by the Johns Hopkins University School of Medicine IRB (IRB# NA_00039294, NCT00005436). Healthy control VRC samples were collected at the National Institutes of Health (NIH) Clinical Center under the Vaccine Research Center's (VRC)/National Institutes of Allergy and Infectious Diseases (NIAID)/NIH protocol “VRC 000: Screening Subjects for HIV Vaccine Research Studies” (NIH 02-I-0127, NCT00031304) in compliance with NIAID IRB approved procedures. All patients from both cohorts gave written, informed consent.

### VirScan assay

VirScan screening was performed as described previously.^5^^,^^6^^,^^14^^,^^15^ Briefly, we used a mid-copy T7 bacteriophage display library spanning the human virome, which consists of 96,099 56-aa peptide tiles that overlap adjacent tiles by 28-aa. BLT5403 *E. coli* (Novagen) was used to expand the library, which was then stored at −80 °C in 10% DMSO. An ELISA was used to quantify IgG serum concentrations (using Southern Biotech capture and detection antibodies, cat# 2040–01 and 2042–05, respectively). Next, 2 μg of IgG was mixed with 1 mL of the VirScan library at a concentration of 1 × 10^10^ pfu (diluted in PBS) for each reaction. Following overnight end- over-end rotation of the phage and serum mixtures at 4 °C, 40 μL of protein A/G coated magnetic beads (Invitrogen catalog numbers 10002D and 10004D) were added to each reaction (20 μL of A and 20 μL of G) which were rotated an additional 4 h at 4 °C. Later, the beads were washed three times and then resuspended in a Herculase II Polymerase (Agilent cat # 600,679) PCR master mix using a Bravo (Agilent) liquid handling robot. This mix underwent 20 cycles of PCR. Subsequently, 2 μL of this amplified product underwent an additional 20 cycles of PCR, during which sample-specific barcodes and P5/P7 Illumina sequencing adapters were added. This product was pooled and then sequenced using an Illumina HiSeq 2500 in rapid mode (50 cycles, single end reads).

### Pairwise differential peptide enrichment analysis

We used pairwise enrichment analysis to identify peptides that were differentially reactive between timepoints. Robust regression of the top 1000, by abundance, Day 1 read counts was used to calculate the ‘expected’ Day 14 read counts. The observed Day 14 read counts minus the expected Day 14 read counts for each peptide was calculated to determine peptide residuals. Peptides were grouped in bins and a standard deviation was calculated between all peptides in each bin. From these binned standard deviations, a linear regression was developed and used to assign each peptide an expected standard deviation. Each peptide's residual was normalized to its expected standard deviation, in order to calculate a 'pairwise z-score'; z-scores > 10 were considered differentially reactive. The Day 1 versus Day 14 read count scatter plots were generated in R 3.6.1 ^16^. Two cases failed our quality control filter, due to poor Day 1 versus Day 14 correlations, and were therefore excluded from further analysis.

### Peptide-virus alignment table

The peptide-virus alignment tables were created as follows. First, all viral genomes, including representative genomes that are in RefSeq and ‘neighbor’ strains that are not in RefSeq, were downloaded on May 2, 2017 in GenBank format from the NCBI Viral Genome Resource.^17^ The host field of the GenBank files and the host column in the NCBI Viral Genome Resource neighbors file were then used to find viral strains that infect humans. Furthermore, all viruses annotated with human host in the Viral-Host Database (v170502) were included.^18^ The human-host annotation was propagated from each viral strain to all strains of the same species. BLAST databases^19^ of nucleotide sequences of the human viruses were created using makeblastdb at sequence, organism and species levels. tblastn v2.2.31+ was run to create peptide-virus alignment tables (parameters: “-outfmt 6 -seg no -max_hsps 1 -soft_masking false -word_size 7 -max_target_seqs 100,000″).

### Network analysis and binomial statistics

AVARDA was developed and implemented in R 3.6.1.^16^ The software reads in a file of hits for each peptide and sample and outputs the list of significant viral infections, along with the associated p-values, assigned counts and peptides to each virus, and other relevant information used in the analysis. AVARDA can be downloaded for general use at https://github.com/drmonaco/AVARDA, where further documentation is provided in a README.

#### Defining the maximal set of nonoverlapping antibody specificities

As described in Module 2, a database of alignments with E values <100 between all peptides in the VirScan library is used to construct undirected network graphs, where each node represents a reactive peptide, and each edge represents a peptide-peptide alignment using the igraph software package. We then calculate a maximal independent vertex set for each network graph. An independent vertex set is a set of vertices in which no two are adjacent and a maximum independent set is the largest possible independent set for a given graph. igraph uses a series of algorithms defined in Tsukiyama et al.^20^ to determine the maximal independent vertex set. Due to computational limitations in running the igraph independence functions, we initially remove the most interconnected peptides iteratively until the most interconnected peptide(s) has 5 alignments, after which we use the igraph independence function to determine the final maximal independent vertex set.

#### Null probabilities for binomial testing

As described in Module 2, when comparing sets of reactive peptides associated with each virus, we use binomial testing to determine the significance of the associations. All binomial tests were calculated in R via the binom.test(k,N,f) function. The null model assumes the peptides aligning to each virus were randomly drawn from the VirScan library. For each virus_k_ with at least three reactive evidence peptide alignments, we perform a binomial test using *N* = total number of reactive peptides, *k* = hits aligning to virus_k_, and the null probability ‘f’, which is the total number of peptide alignments to virus_k_, divided by the total number of peptides in the VirScan library. Viruses with less than three reactive evidence peptide alignments are assumed insignificant. Evidence alignments are defined as bit score alignments ≥ 80, an adjustable user-defined threshold. As the bit score threshold is increased, fewer peptides are considered evidence for a given virus, reducing sensitivity but increasing specificity. Increasing the threshold will also lead to fewer indistinguishability tags, as fewer peptides will be associated with multiple viruses.

#### Shared peptide alignments

All pairs of viruses that share reactive peptide alignments are evaluated as follows in Module 3. First, a binomial test is used to evaluate the non-shared virus_k_ and virus_m_ peptides, in order to weigh the evidence for each infection on its own. For this test where virus_k_ is compared against virus_m_, k is the number of non-shared virus_k_ peptides, *f* = (number of evidence peptides aligning to virus_k_ but not virus_m_) / (size of library – shared alignments between virus_k_ and virus_m_). Similarly, *N* = number of total reactive peptides – shared virus_k,m_ peptides. A similar binomial test is performed where virus_m_ is evaluated on its own. To determine significance, we set a p-value threshold of *p* < 0.05 and require that the number of non-shared evidence peptide alignments is at least 3. These parameters can be adjusted depending on the desired stringency of the analysis. As this threshold is lowered, AVARDA's detection sensitivity may increase but at a cost of reduced specificity.

#### Viral comparisons, shared alignment removal, and indistinguishability tags

N is first initialized to the number of reactive, independent peptides. All viruses are then ranked in order of significance based on binomial testing of each virus's reactive evidence peptide alignments as described in Module 2. Next, the most significant, "index" virus is compared to every other virus which shares peptide alignments as described in Module 3. In these comparisons, if either virus is found to have a statistically significant number of non-shared peptides while the other does not, the shared alignments are removed from the latter virus (case i in Figure 2**c**). If both viruses are found to have statistically significant numbers of non-shared peptides, the shared alignments remain associated with both viruses, thus contributing to the significance of both infections (case **ii** in Figure 2**c**). If neither virus is found to have a statistically significant number of non-shared peptides, binomial testing is performed on non-shared and shared peptides to determine whether there is sufficient evidence for infection by at least one of the two viruses. If the viruses have a significant number of shared alignments, a numerical “indistinguishability tag” is annotated to the results to indicate that the algorithm was unable to accurately distinguish between infection by one or both of the viruses (case **iii** in Figure 2**c**). Once the pairwise comparisons with the most significant virus have been completed, the index virus is then removed from consideration and N is decreased by the number of peptides that were uniquely assigned to the index virus. This procedure of updating N thus removes peptides associated with significant infections from consideration in subsequent tests of peptides using the null model. The *p*-values for each remaining virus are recalculated, and the new top ranking index virus_k_ is selected for the next round of comparisons until all virus_k,m_ pairs have been evaluated. Finally, all p-values are adjusted for multiple hypothesis testing using the Benjamini-Hochberg procedure considering all infections. Ancillary relevant data are also printed to file.

### Clinical antibody tests

All herpes simplex virus, varicella zoster virus, cytomegalovirus and Epstein-Barr virus antibody testing were carried out on a Diasorin Liason®, which uses chemiluminescent immunoassay technology, at the Johns Hopkins Pathology Core facilities. All other serological analyses were performed by Quest Diagnostics; human herpesvirus 6 and 8 antibodies were analyzed via immunofluorescence assay technology and human parainfluenza virus 3 antibodies were analyzed via complement testing. Enterovirus testing was not done due the large volume of sera required per test. Due to the limited volume of sera, all samples were diluted 1:10 prior to testing. Final results were interpreted by a clinical pathologist.

### Role of funding source

This work was made possible by support from the National Institutes of Health (NIH), the US Army Research Office, the Singapore Infectious Diseases Initiative (SIDI), the Singapore Ministry of Health's National Medical Research Council (NMRC) and the Singapore National Research Foundation (NRF). The funders had no role in the design of this study, data collection, data analyses, data interpretation, or writing of this manuscript.

## Results

### Acute versus convalescent VirScan profiles

**Figure 1:**
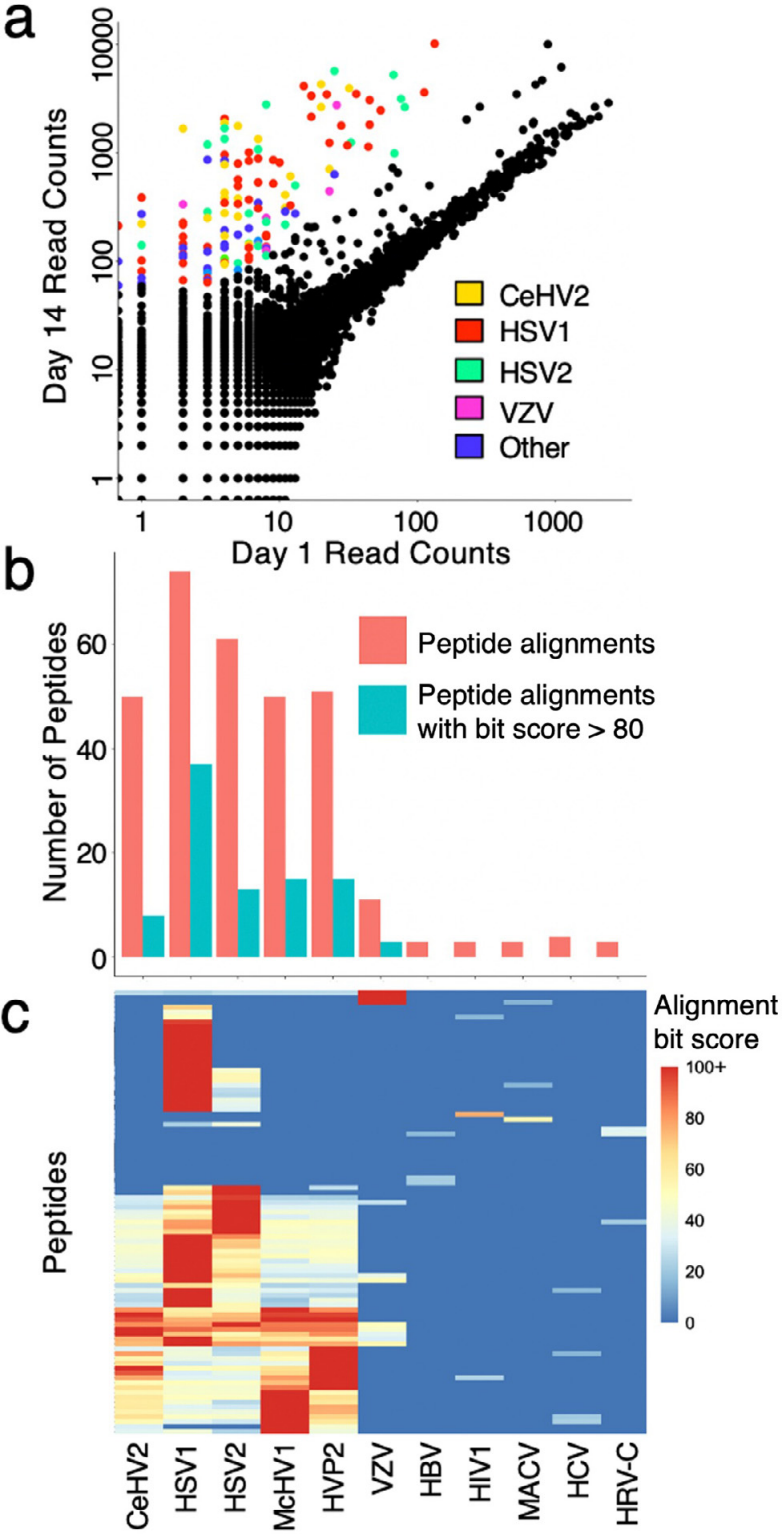
Acute versus convalescent VirScan analysis of an HSV1 encephalitis patient. (a) VirScan read counts plotted for Pt1 at day 14 versus day 1 (hospital admission). Peptides of pairwise z-score >10 are colored according to the virus they were designed to represent. (b) Total reactive peptides aligning to each virus are shown in red. Numbers of reactive evidence peptides (tblastn bit score >80) for each virus are shown in blue. Virus species as indicated below in c. (c) Clustered heatmap of the reactive peptide alignments, colored by alignment bit score. CeHV2, Cercopithecine herpesvirus 2; HSV1, Herpes simplex virus 1; HSV2, Herpes simplex virus 2; McHV1, Macacine alphaherpesvirus 1; HVP2, Papiine herpesvirus 2; VZV, Varicella-zoster virus; HBV, Hepatitis B; HIV1, Human immunodeficiency virus 1; MACV, Machupo virus; HCV, Hepatitis C; HRV-C, Human rhinovirus C.

The quality of the VirScan library was assessed via sequencing to a depth of 360-fold coverage. We detected sequences from 99.7% of the 106,678 unique clones in the VirScan library, and found they were drawn from a relatively uniform distribution (Figure S1). We performed VirScan analysis on paired serum samples from an encephalitis cohort, collected at hospital admission and fourteen days later. To quantify changes in the antiviral antibody repertoire over time, we utilized a pairwise z-score based approach (Methods). Changes in antibody binding over time were considered significant when the day 0 versus day 14 pairwise z-scores were greater than 10. Figure 1**a** shows the results of one patient's (Pt1) pairwise VirScan analysis. Nucleic acid testing had previously diagnosed a herpes simplex virus 1 (HSV1) infection in this individual, consistent with the predominance of reactive peptides designed to represent alphaherpesviruses. Notably, antibodies from this patient with HSV1 encephalitis showed binding to 15 distinct peptides that were designed to represent HSV2, highlighting the importance of considering antiviral antibody cross-reactivity when interpreting VirScan data.

### AVARDA Module 1: enumerating peptide-virus associations

In previous studies, each VirScan peptide has been defined simply by the specific viral protein it was intended to represent. However, in order to exhaustively consider sequence similarity and thus potential for antibody cross reactivity, we have constructed a comprehensive genomic database of all known human viral species. To exclude any potential open reading frame annotation bias, all VirScan peptide sequences were aligned via tblastn against a protein sequence database formed by translating these viral genomes in all 6 reading frames.^21^ The relationship of all VirScan peptides to all human viruses can thus be represented as a matrix of alignment scores. This alignment score matrix naturally features a broad distribution of alignment strengths. Here, we dichotomize alignments by a bit score threshold of 80, considering alignments greater than 80 to be potential “evidence” for an infection, while alignments below 80 are considered potentially “cross-reactive” alignments. Evidence peptides can be used in support of an infection, whereas cross-reactive alignments can be used in support of a “false positive” reactivity. Figure 1**b** compares the number of total alignments versus evidence alignments to each virus associated with at least 3 reactive peptides. The matrix of alignment scores associated with Pt1’s peptide reactivities is provided as a clustered heatmap in Figure 1**c**.

Figure 1**c** illustrates that most of Pt1’s reactive peptides align with multiple viruses. Also apparent is that most of these alignments are shared among the human alphaherpesviruses. As expected, given the diagnosed HSV1 infection, however, almost every single peptide that aligns with an alphaherpesvirus, also aligns with HSV1.

### Module 2: defining the maximal independent vertex set of reactive peptides associated with each virus

**Figure 2:**
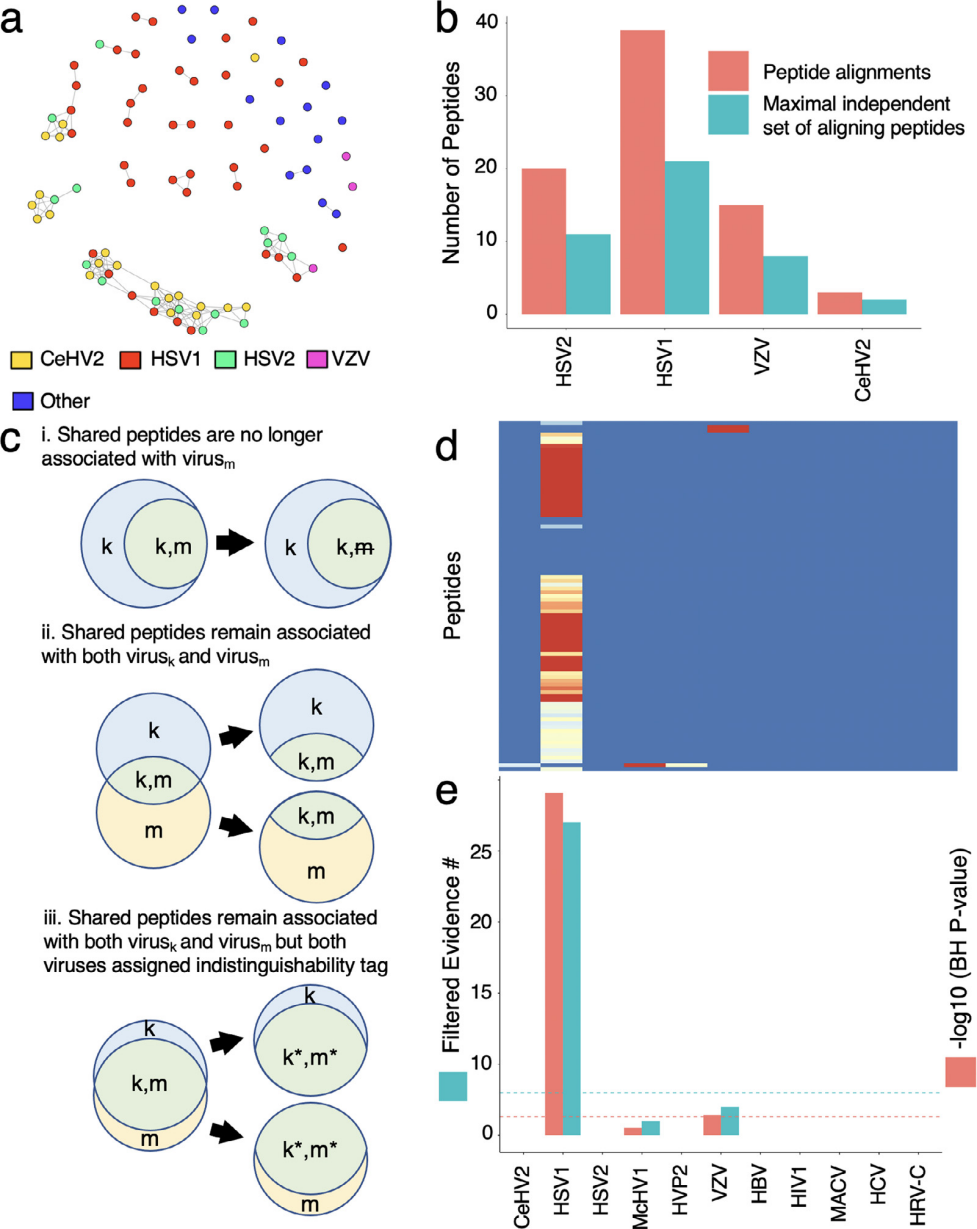
Associating peptides and viruses using alignment and deconvolution. (a) Network graph depicting sequence similarities among the reactive set of peptides shown in Figure 1a. Nodes represent peptides and edges are drawn between peptides that share a blastp alignment with an E value of at most 100. (b) Numbers of Pt1’s reactive peptides associated with the viruses shown in 2a. Total number of reactive peptides representing each virus by design are shown in red. Numbers of reactive peptides to each virus after filtering for independence are shown in blue. (c) Schematic representing three possible outcomes of shared peptide alignments between two viruses: (i) single infection by virus_k_ can account for all reactive peptides that align to virus_m_, (ii) infections caused by both virus_k_ and virus_m_, and (iii) an infection by virus_k_ and/or virus_m_ which cannot be distinguished. (d) The peptide alignment heatmap of Figure 1c after removal of alignments determined to be cross-reactive. (e) Numbers of independent evidence peptides and –log10 BH corrected P-values for each virus after consideration of cross-reactivity via Module 3.

The VirScan library was designed to represent the proteins of the human virome as overlapping 56 amino acid peptide tiles. Due to the 28 amino acid overlaps, as well as homologous sequences from related proteins, a large amount of epitope redundancy exists within the library. To account for these sequence similarities, we performed a blastp alignment that compares each peptide to all others in the library. Using these alignment results, we built a database of all VirScan peptide-peptide alignments at an E value threshold of 100, a threshold we previously established for defining potential cross-reactivity.^22^ This database can thus be used to construct a network graph that depicts the sequence similarities among reactive peptides. Figure 2**a** illustrates the sequence similarities among Pt1’s longitudinally reactive peptides.

To determine the breadth of the antibody response, which we define as the largest number of reactive peptides that do not share any sequence similarities (potential cross-reactivities), we utilize a concept from network graph theory called the maximal independent vertex set. An independent set is defined as a set of vertices in a graph, such that no two are connected. A maximal independent set is an independent set that is not a subset of any other independent set. To calculate the maximal vertex set, we utilized the algorithm described by Tsukiyama et al.,^20^ which is implemented by the R package igraph (Methods). By separately calculating the maximal independent set for each virus, we can estimate the response breadth (clonality) directed at each virus. The total (“unfiltered”) and maximally independent (“filtered”) evidence peptides for Pt1’s potential viral infections are provided in Figure 2**b**. Module 2 additionally provides a probabilistic assessment of infection for each virus via binomial testing; for each virus the ratio of reactive independent evidence peptides over all reactive independent peptides is compared against the fractional representation of that virus in the VirScan library.

### Module 3: deconvoluting viral reactivity profiles

Conceptually, by associating all reactive peptides with all possible viral infections, Module 2 increases the sensitivity, but decreases the specificity of the VirScan assay. Module 3 therefore seeks to weigh the evidence for each infection on its own, given the relationship of all evidence peptides to all viruses. The most significant virus (‘virus_k_’), as determined by Module 2, is iteratively compared with viruses that share peptide alignments (‘virus_m_’); viruses must have at least 3 reactive independent evidence peptides to be evaluated. Peptides are considered as either shared or non-shared: shared peptides align to both virus_k_ and virus_m_ and non-shared peptides align exclusively to either virus. Peptides that are cross-reactive for one virus and evidence for the other are considered shared. Detailed in the Methods, the non-shared peptides in a given virus_k_-virus_m_ comparison are first evaluated for significance using a binomial test.

The three possible outcomes for each virus_k_-virus_m_ comparison are illustrated in Figure 2**c**: In scenario **i**, the number of non-shared virus_k_ evidence peptides is significant, while the number of non-shared virus_m_-evidence peptides is not significant, suggesting that reactivity of the shared peptides can be attributed to infection only with virus_k_. In this case, the peptides aligning to both viruses are no longer associated with virus_m_. In scenario **ii**, the number of non-shared evidence peptides are significant for both viruses, suggesting that the observed reactivities can be attributed to infections by both virus_k_ and virus_m_. In this case, we allow the shared evidence peptides to serve as evidence for each infection separately. In scenario **iii**, the number of shared evidence peptides is significant, but the number of non-shared evidence peptides associated with either virus alone is not significant. In this scenario we assume the reactivities are most likely due to an infection with at least one of the two viruses, but there is insufficient evidence to distinguish an infection from *either one* or *both* viruses. Both viruses are therefore assigned a unique numeric tag to indicate that their potential infections could not be distinguished. After the probabilistic assessment of virus_k_’s shared peptide-virus associations, all peptides associated exclusively with virus_k_ are removed from further consideration. Module 2 is called again to provide updated p-values for each remaining virus, and the process is repeated until all virus pairs with shared peptide alignments have been analyzed. Upon completion, Module 3 will have conservatively removed false positive viral associations, while having retained each peptide's association with the most likely infection(s), including indistinguishable infections.

Figure 2**d** shows the same alignment matrix of Figure 1**c** but updated by Module 3 to remove the potential peptide-virus associations that are no longer considered evidence of an infection. The final binomial test p-value for each virus is then adjusted for multiple hypothesis testing using the Benjamini-Hochberg (“BH”) procedure ^23^ (p_BH_-value). For Pt1, nearly every longitudinally reactive peptide is only considered likely to be associated with an HSV1 infection. If we require at least 3 evidence peptides to support an infection, and a p_BH_-value of below 0.05, Pt1 is determined to have a single infection, HSV1 (p_BH_-value = 8.43 × 10^−25^). The final -log10(p_BH_-values) and the number of independent evidence peptides associated with all viruses from Figure 1**b** are provided in Figure 2**e**. Module 3 generates several useful files for understanding how each peptide was evaluated during the course of its analysis.

### AVARDA for unbiased diagnosis of viral encephalitis

**Figure 3:**
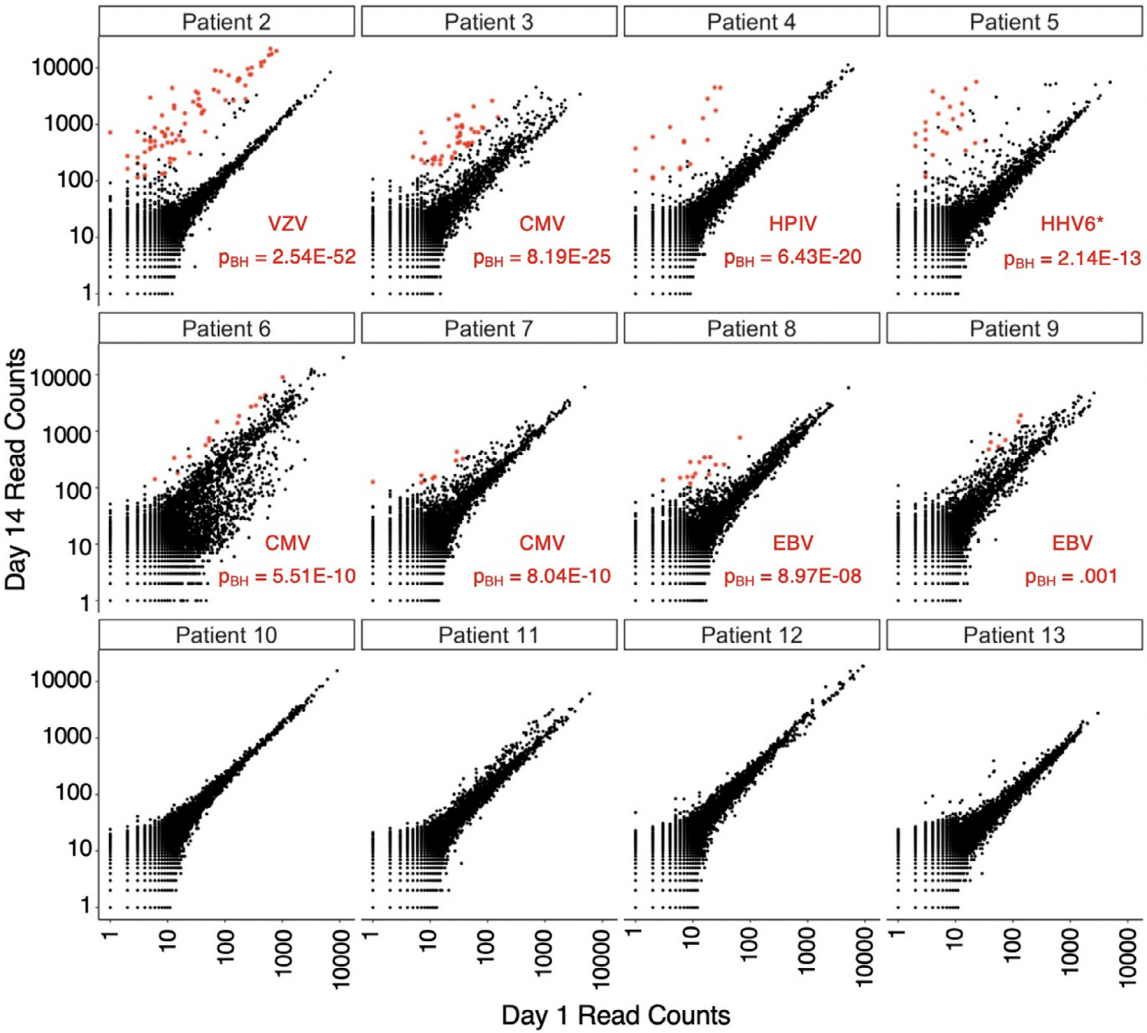
AVARDA analysis of acute versus convalescent antibody reactivities from twelve patients with encephalitis. Each plot indicates the read counts from the day 14 VirScan profile (y-axis) plotted against the day 1 VirScan profile (x-axis). Peptides with pairwise reactivity z-scores >10 that were associated with a significant infection determined by AVARDA are marked in red.

The AVARDA framework we established using Pt1’s longitudinal data was subsequently applied to 76 additional encephalitic patients’ sera, 40 with confirmed non-viral clinical diagnoses. Pairwise analysis of VirScan data followed by AVARDA generated no significant viral results for any of the 40 non-viral cases (not shown). Of the other 36 individuals, eight (in addition to Pt1) had received diagnoses of viral infection based on nucleic acid testing (Pt2, Pt3, Pt5, Pt7, Pts10–13; Table 1); the remainder were undiagnosed and treated as potential viral encephalitis. The summarized AVARDA findings, as well as the nucleic acid and confirmatory clinical serological test results, are provided in Table 1; patients not diagnosed with viral infection based on nucleic acid test or by AVARDA^23^ are not included for brevity. Requiring at least three independent reactive peptides and a p_BH_-value < 0.05, AVARDA proposed infections in eight patients (Pts2–9). The paired longitudinal VirScan data are provided in Figure 3. Three of the eight nucleic acid test positive cases (Pt2, Pt3 and Pt7) corresponded precisely with the infections proposed by AVARDA. Four of the eight nucleic acid test positive cases had no infections detected by AVARDA; these cases were diagnosed with HSV (Pt10), enterovirus (Pt11), and varicella-zoster virus (VZV, Pt12 and Pt13); the clinical tests were unable to distinguish between HSV1 and HSV2 in Pt10, and unable to detect which species of enterovirus in Pt11. Results for Pt5 were discordant with AVARDA, having a PCR positive test result for cytomegalovirus (CMV), whereas AVARDA proposed a roseolavirus infection (10 independent HHV6 peptide reactivities, p_BH_ = 2.14 × 10^−13^). Closer investigation of Pt5’s clinical history revealed previous Ganciclovir treatment for CMV infection, suggesting a long-term prior infection. Consistent with this, AVARDA detected significant CMV antibodies at both day 1 and 14, but their reactivity was unchanged over time. Importantly, a clinical antibody test agreed with the results of AVARDA, measuring increased HHV6-specific IgG in the convalescent serum. Of the 9 AVARDA predictions, 7 were confirmed by either nucleic acid or clinical antibody testing. The remaining two had negative nucleic acid results and insufficient serum for further antibody testing.

**Table 1:** Summary of AVARDA results from the encephalitis cohort. The ‘Virus’ column indicates the viral infection identified by AVARDA or nucleic acid test. The ‘BH p-value’ column represents the multiple test-corrected AVARDA p-values. ‘Total Sample Hits’ refers to the total number of peptides found to be increasing in reactivity during the two-week interval. ‘Total Filtered Evidence Hits’ indicates the number of reactive peptides associated with the virus after AVARDA analyses. ‘Filtered N-rank #’ indicates the total number of reactive peptides considered by AVARDA for a given infection. Nucleic acid or serologic test results are indicted in the last two columns. '–' indicates that a specific test was not conducted. ‘*’ HHV6A and B infections were both indistinguishably identified but reported as HHV6. ‘+’ indicates serology testing that did detect virus specific IgG but it was unchanged between timepoints.

Pt Number	Virus	BH P-value	Total Sample Hits	Total Filtered Evidence Hits	Filtered N-rank #	Nucleic Acid Test	Serology Test
1	HSV1	8.43 E-25	91	22	37	HSV1	–
2	VZV	2.54 E-52	93	34	43	VZV	VZV
3	CMV	8.19 E-25	42	18	19	CMV	–
4	HPIV3	6.43 E-20	22	8	10	–	–
5	HHV6*	2.14 E-13	41	10	18	CMV	HHV6
6	CMV	5.51 E-10	25	10	19	–	CMV
7	CMV	8.04 E-10	14	8	11	CMV	–
8	EBV	8.97 E-08	16	5	7	–	EBV
9	EBV	0.001	15	3	8	–	–
10	HSV	1	0	0	0	HSV	HSV+
11	Enterovirus	1	0	0	0	Enterovirus	–
12	VZV	1	0	0	0	VZV	VZV+
13	VZV	0.016	1	1	1	VZV	VZV

Single timepoint AVARDA analyses (Table S1) and clinical antibody testing both detected high but unchanging HSV and enterovirus specific IgG levels in Pt10 and Pt11, respectively. This suggests that either (i) these were chronic or prior infections potentially unrelated to their encephalitis, (ii) these patients’ initial sera were collected too late in the course of their illness to see longitudinal antibody changes, or (iii) VirScan did not detect the changing antibody specificities/titers. Clinical antibody testing detected increasing VZV-specific IgG levels in Pt13, which was not detected by AVARDA using longitudinal analysis; however, AVARDA using single timepoint analysis did detect VZV reactivity at both timepoints for Pt12 and Pt13 (in both cases a small number of additional reactivities were detected at the second time point). Pt13’s longitudinal VirScan results revealed several VZV aligning peptides that appeared to be increasingly reactive over time, but the changes were below our threshold. This finding indicates that our default threshold may be too stringent in some cases. In individuals with undiagnosed encephalitis, AVARDA detected infections with HPIV3 (Pt4), CMV (Pt6), and Epstein-Barr Virus (EBV, Pt8 and Pt9). Confirmatory clinical serology detected increasing viral IgG for two of these cases (CMV for Pt6 and EBV for Pt8). We were unable to conduct clinical antibody tests for Pt4, Pt9 and Pt11, due to insufficient remaining serum volume. Overall, AVARDA analyses of longitudinal VirScan data were largely concordant with clinical test results. AVARDA analyses of single timepoint VirScan data may provide additional information in some cases, but doesn't distinguish active from latent or historic infections.

### AVARDA analysis of a longitudinal T1D cohort

The post infectious sequalae of antiviral immune responses are believed to precipitate a number of autoimmune diseases,^24^ including type 1 diabetes (T1D). Longitudinal cohort studies provide a powerful approach for causally linking environmental exposures with development of disease.

**Figure 4:**
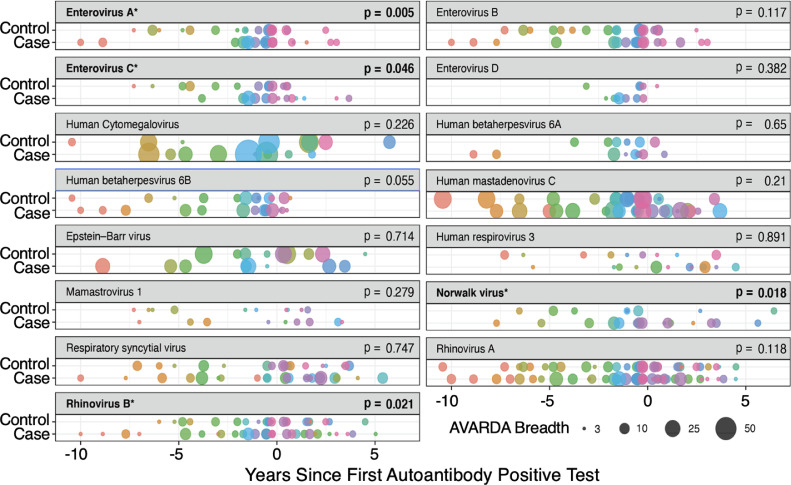
Longitudinal AVARDA analysis of patients with type 1 diabetes. The x-axis is patient age minus the age at first autoantibody positive test (or corresponding matched control age). Each infection deemed significant with AVARDA (adjusted p-value less than 0.05), is plotted at the midpoint of the sampling interval. Infections are colored by patient and point size corresponds to the antibody response breadth (T1D cases: *n* = 14, bottom; controls: *n* = 14, top). Differences in antibody breadth and normalized ages, among the cases versus the controls, were assessed for each virus via one-sided Wilcoxon signed-rank test. Only infections identified with AVARDA prior to first autoantibody positive test were evaluated. For individuals with multiple infections by the same virus, the largest response breadths were used for breadth comparisons and the earliest and latest age at infection were, separately, used for normalized age comparisons. There were no significance differences (*p* < 0.05) in time since seroconversion for any virus. Four viruses (Enterovirus A, Enterovirus C, Norwalk virus, and Rhinovirus B) had higher antibody response breadths in the cases (*p* < 0.05, one-sided Wilcoxon signed-rank test, p-values shown in each plot header).

We used VirScan/AVARDA to profile sera collected every two to six months from 14 children at high genetic risk for T1D followed prospectively until diagnosis of T1D^11^^,^^12^ Each of the 14 T1D cases had a corresponding high genetic risk control, matched for age, sex, and HLA-DR/DQ, who has not developed T1D or islet autoantibodies. In order to perform a uniform analysis across this longitudinal cohort, we considered sample collection time points as relative to the first islet autoantibody in the cases and the corresponding ages in the controls. We restricted subsequent analyses to the 15 viruses that were detected in at least 10 individuals prior to development of the first autoantibody or matched control timepoint. The breadths of the immune responses are visualized by virus species for each individual as a function of autoantibody-relative time (Figure 4). Two metrics of these infections were compared between cases and controls: response breadths and autoantibody-relative infection times. The distribution of breadths and infection times were compared using a one-sided Wilcoxon signed-rank test without multiple test correction. Four virus species showed higher antibody response breadths in the cases versus the controls (*p*-< 0.05): Enterovirus A, Enterovirus C, Norwalk virus, and Rhinovirus B. No viruses were associated with relative time to autoantibody development. A recent metagenomic sequencing-based study by Vehik et al.^25^ detected an association between enterovirus B and the development of T1D. In our study, however, AVARDA detected the strongest antibody association with Enterovirus A, and a weaker association with Enterovirus C. This difference may be due to inaccurate deconvolution of the highly cross-reactive enteroviral antibodies or to differences in the pathogenic enteroviral exposure profiles between the two cohorts.

### AVARDA analysis of a cross-sectional lupus cohort

Systemic Lupus Erythematosus (SLE, lupus) is a poorly understood multisystem autoimmune disorder, which is associated with the development of several autoantibodies targeting nuclear antigens, most commonly double-stranded DNA.^26^ Lupus susceptibility has been linked most strongly to HLA alleles, complement components and the antibody Fcγ receptors, implicating antibody effector functions in the disease pathophysiology.^27^ Gene expression studies of peripheral blood have identified a type I interferon signature, which corresponds with elevated levels of circulating interferon alpha.^28^ A growing body of evidence has linked EBV infection with the development of lupus.^29^^,^^30^ Like all human herpes viruses, EBV has a double stranded DNA genome and can activate the type I interferon system.

**Figure 5:**
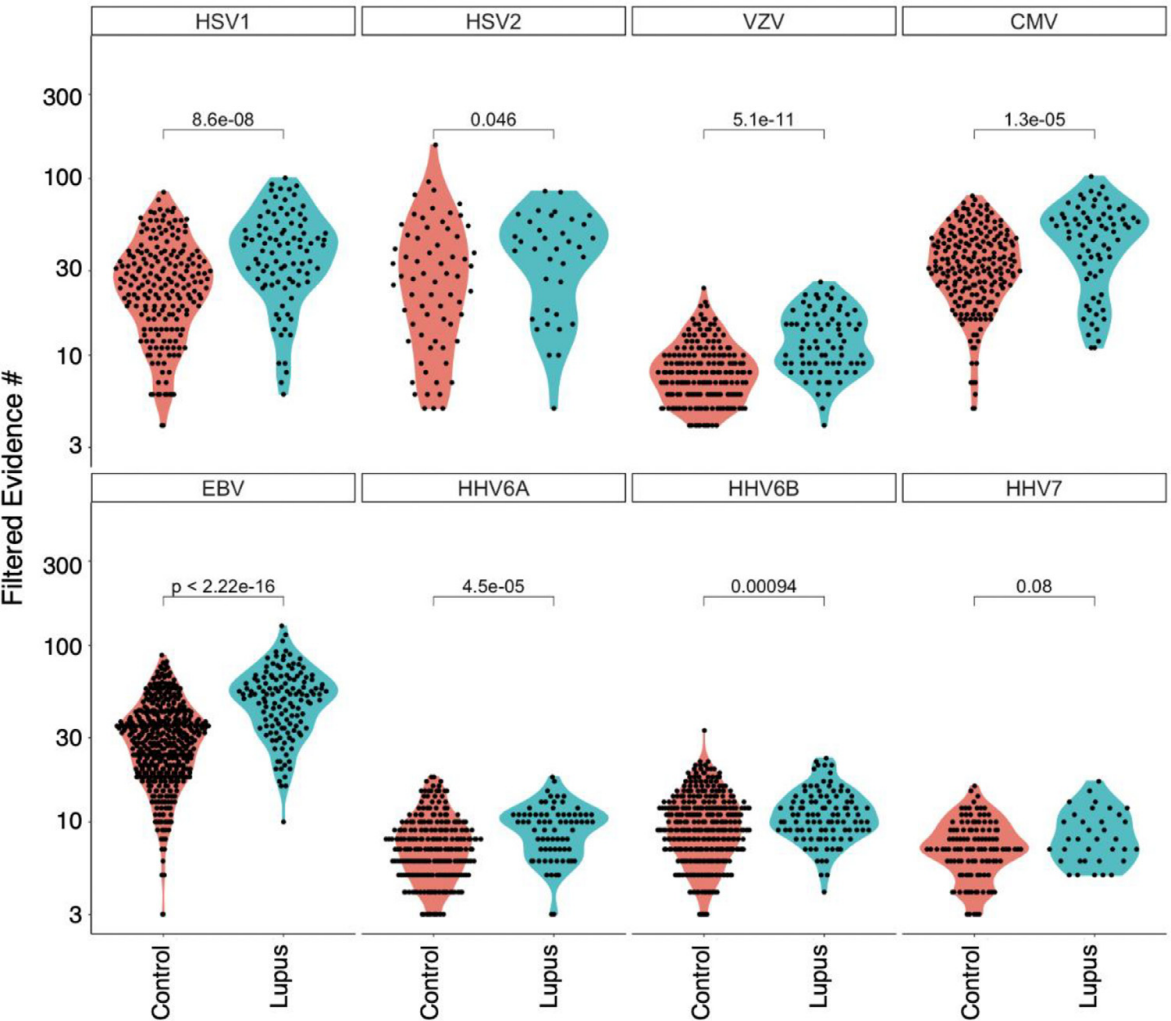
Cross-sectional AVARDA analysis of patients with lupus. Violin plots evaluating antibody breadth between all lupus samples (*n* = 142) and controls (*n* = 501) for HHV1–7. Antibody breadths were only considered when AVARDA determined an infection at p_BH_ < 0.05. Significant differences were observed for HSV1, HSV2, VZV, CMV, EBV, HHV6A and HHV6B (two sided Wilcoxon signed-rank test).

We used VirScan to perform an unbiased assessment of viral antibodies in a cross-sectional cohort of 142 non-Hispanic white lupus patients and 501 controls matched for ethnic background. We assessed both prevalence and response breadth to all human viruses **(**Figure 5**).** We detected a robust increase in EBV response breadth in lupus patients compared to controls (p < 2 × 10^−16^). Increased response breadths were also notable for HSV1, VZV, CMV, HHV6B and HHV6A. We additionally noted an increase in seroprevalence for HSV1, HSV2, VZV and EBV in lupus patients compared to controls **(**Table 2**)**. In a Black cohort (lupus *n* = 118, controls *n* = 159), prevalence and response breadth associations were similar to the non-Hispanic white cohort. However, we did not observe a difference in prevalence for EBV (cases and controls were uniformly seropositive). These data are consistent with prior studies that have found lupus patients to have increased prevalence of CMV and HSV1,^31^ increased antibody titer to EBV^32^ and VZV,^33^ and elevated HHV-6 and EBV viral load.^34^ Here we report a previously undescribed expansion of the response clonality targeting many human herpesviruses.

**Table 2:** Summary of AVARDA results from the lupus cohorts. A non-Hispanic white cohort (lupus *n* = 142, control *n* = 501) and a Black cohort (lupus *n* = 118, control *n* = 159) were analyzed independently. The ‘Virus’ column indicates the viral infection identified via AVARDA. Lupus Positive % and Control Positive % refer to the fraction of lupus and control patients that were determined seropositive by AVARDA. ‘Average Breadth’ indicates the mean antibody response breadths among seropositive individuals. ‘SD’ indicates the standard deviation of the antibody response breadths. ‘Positivity P-val (BH)’ was determined via a two-sided fisher's exact test (BH corrected). ‘Breadth P-val (BH)’ was determined via a two-sided Wilcoxon signed Rank test (BH corrected).

Virus	Ethnicity	Lupus Positive %	Control Positive %	Seropositivity BH P-val	Control Average Breadth	Lupus Average Breadth	Control SD	Lupus SD	Breadth BH P-val
HSV1	Black	0.76	0.6	**0.012**	33.295	48.789	16.011	22.134	**2.00 E-06**
HSV1	Caucasian	0.63	0.4	**1.20 E-05**	28.02	42.135	16.205	21.987	**2.29 E-07**
HSV2	Black	0.57	0.41	**0.021**	33.138	57.985	19.166	30.327	**1.33 E-06**
HSV2	Caucasian	0.26	0.134	**0.0028**	32.896	39.889	26.732	21.154	0.052
VZV	Black	0.51	0.29	**0.0022**	9.13	12.667	4.787	5.164	**6.72 E-05**
VZV	Caucasian	0.54	0.36	**8.50 E-04**	8.566	12.803	3.528	5.07	**2.04 E-10**
CMV	Black	0.84	0.66	**0.0036**	36.752	55.455	16.757	23.474	**9.60 E-09**
CMV	Caucasian	0.51	0.42	0.089	34.9	47.486	15.975	21.887	**2.60 E-05**
EBV	Black	0.99	0.97	0.53	32.219	53.821	14.624	21.606	**1.78 E-15**
EBV	Caucasian	0.96	0.89	**0.015**	31.062	51.404	15.562	21.059	**1.78 E-15**
HHV6A	Black	0.51	0.47	0.72	8.933	9.083	4.16	2.993	0.35
HHV6A	Caucasian	0.52	0.45	0.17	7.796	9.338	3.291	3.026	**7.20 E-05**
HHV6B	Black	0.76	0.78	0.77	10.218	11.344	4.662	3.889	**0.018**
HHV6B	Caucasian	0.72	0.66	0.3	10.027	11.396	4.415	3.901	**1.25 E-03**
HHV7	Black	0.18	0.23	0.48	7.595	10.095	3.158	3.506	**0.0096**
HHV7	Caucasian	0.25	0.23	0.57	7.513	8.657	2.709	3.067	0.08

## Discussion

The diagnostic and epidemiologic utility of antibody analyses has been limited in large part due to the difficulty in accounting for antibody cross reactivities among closely related organisms.^10^ Coupling of pan-viral peptide level profiling technologies with an analytical framework that systematically accounts for cross reactivity can therefore expand the utility of serologic profiling. Our current study has demonstrated that AVARDA can be used to accurately deconvolute unbiased VirScan data.

We have examined the performance of AVARDA in diagnosing viral encephalitis, a setting in which it is often challenging, yet critical, to distinguish infectious from autoimmune etiologies.^28^^,^^29^ As such, pan-viral IgG analysis may provide information complementary to nucleic acid testing, thus improving diagnostic sensitivity for low abundance or unexpected viruses, and for distinguishing active from latent infections. These analyses can also identify peptide reactivities that improve the performance of traditional serological testing formats. In this study, combining VirScan/AVARDA analysis with traditional diagnostic methods increased the rate of diagnosis by 44.4%. Pan-viral IgM analyses, while not explored here, may also be informative in differentiating acute from chronic infections, without the need for pairwise comparisons. Like any antibody test, however, VirScan/AVARDA still requires the development of an adaptive immune response, which takes valuable time and may not occur in patients with suppressed immune systems. Furthermore, AVARDA only interprets antibody reactivities identified by VirScan and, as such, cannot detect antibodies targeting viruses absent from the VirScan library and may be less sensitive in detecting antibodies targeting viruses that tend to elicit antibodies primarily to conformational epitopes.

We have additionally explored the utility of using AVARDA to link viral infections with two autoimmune diseases, T1D and in lupus. In both cases, AVARDA identified a set of viral associations that expand upon prior studies. Our key findings were related to antibody breadths, which reflect the polyclonality – and thus the robustness–of the immune response. Enhanced polyclonality could reflect elevated antigenic exposure (due to increased viral load or persistence) or rather a more intense inflammatory milieu. Adaptation of the maximal vertex set to antibody clonality thus provides a powerful summary statistic for epidemiological studies.

We propose four general areas in which future iterations of AVARDA could more effectively incorporate VirScan information. First, peptides reactive above a threshold are considered binary hits. If the contribution of each peptide to the final results were to be weighted according to the magnitude of its reactivity score, the confidence of the associated infection(s) could potentially be calculated more accurately. Second, reactive sets of peptides that share sequence homology are currently treated as a single independent data point. However, the confidence associated with the reactivity of related peptides is, conceptually, higher than for single peptides reactive in isolation. If Module 2 attributed greater confidence to these peptide clusters, the sensitivity of AVARDA may be increased. Third, it may be possible to develop a statistical test that can distinguish among infections which are currently found to be indistinguishable by AVARDA. One can envision pairwise comparisons of homologous peptides that might discern the more likely of two highly related species (or even strains), when there is a consistent preference for antibody recognition of one organism's peptides over those of another. If such an approach were successful, it would enable expanding the taxonomic reference database beyond the virus species level, potentially enhancing the specificity of AVARDA. Even at the species level, AVARDA frequently cannot distinguish highly interrelated viruses, particularly enteroviruses and adenoviruses in our experience. Finally, it may be possible to utilize structural similarity, rather than sequence-based alignment, for associating peptides with viruses and identifying potential cross-reactive epitopes. Recent advances in protein folding may soon make such an approach computationally tractable.^35^^,^^36^ We expect that these, and other iterations not contemplated here, will enhance the performance of future versions of AVARDA.

The conceptual paradigm underlying AVARDA may find applications beyond analysis of species-level VirScan data. For example, AVARDA could be adapted to data from a library focused on multiple strains of a specific virus, potentially considering differential strengths of reactivity to strain-defining variant peptides. In any study involving library redundancy and potential antibody cross reactivity, any or all of AVARDA's modules may enable deconvolution of the data. For instance, libraries of bacterial, parasitic, or fungal pathogen peptidomes may be analyzed analogously to VirScan data. Additionally, incorporating the conceptual framework of AVARDA into the design of peptide libraries may enhance their ultimate discriminatory power. Beyond peptide library screening, full-length protein library screening (e.g., via protein microarrays, PLATO, MIPSA etc.) may also benefit from AVARDA's consideration of shared sequences and potential cross-reactivity.

PhIP-Seq analysis of the human proteome is often performed in conjunction with VirScan. In the setting of undiagnosed encephalitis, for example, a combined human proteomic and pan-viral analysis could prove particularly informative. Bayesian interpretation of the joint data sets could potentially be performed under an assumption of either autoimmunity or infection, which may further improve diagnostic sensitivity. Viral infections associated with specific autoantibody reactivities may also be readily detected in longitudinal and cross-sectional studies. AVARDA is therefore an important new tool for linking human health with environmental exposures in a variety of settings.

### Contributors

D.R.M., S.V.K., and H.B.L. conceived the project, designed the algorithm and wrote the manuscript. D.R.M. additionally developed the software for analysis of Illumina sequencing data and implemented AVARDA in R. F.B. constructed the viral genome database and generated the virus-peptide alignment matrix. L-F.W., D.E.A., L.V., K.T. and W.C. provided the encephalitis cohort samples, conducted sample selection, preliminary testing and provided useful insight. K.K. provided valuable guidance for statistical analyses. M.C. interpreted all clinical antibody tests. M. Ro. provided the VRC control cohort. M.Re. and K.W. provided the T1D longitudinal samples as well as valuable information on the patient's vaccine history. M.P. and D.W.G. provided the Hopkins Lupus Cohort samples and clinical data. D.R.M., S.V.K., and H.B.L. verified the underlying data. All authors have read and approved the final version of the manuscript.

## Declaration of interests

H.B.L. is an inventor on a licensed patent describing the VirScan technology, is a founder of Portal Bioscience, Alchemab, and ImmuneID, and serves as an advisor for TScan Therapeutics. The other authors have no potential conflicts of interest to report.

## Appendix Supplementary materials

Image, application 1
