# Supplementary material for: Deconvoluting virome-wide antibody epitope reactivity profiles
Source: eBioMedicine. 2021 Dec 16;75:103747. doi: 10.1016/j.ebiom.2021.103747 (PMC8688874; doi:10.1016/j.ebiom.2021.103747)
Supplement: Supplementary file 1 [file mmc1.docx]

Supplementary Material for:

Deconvoluting virome-wide antibody epitope reactivity profiles

Daniel R. Monaco^1,^*, Sanjay V. Kottapalli^1,^*, Florian P. Breitwieser^2^, Danielle E. Anderson^3^, Limin Wijaya^4^, Kevin Tan^5^, Wan Ni Chia^3^, Kai Kammers^6^, Patrizio Caturegli^1^, Kathleen Waugh^7^, Mario Roederer^8^, Michelle Petri^9^, Daniel W. Goldman^9^, Marian Rewers^7^, Lin-Fa Wang^3^, & H. Benjamin Larman^1,†^

^1^Institute of Cell Engineering, Division of Immunology, Department of Pathology, Johns Hopkins School of Medicine, Baltimore, MD, USA.

^2^Center for Computational Biology, McKusick-Nathans Institute of Genetic Medicine, Johns Hopkins University School of Medicine, Baltimore, Maryland 21205, USA.

^3^Programme in Emerging Infectious Diseases, Duke-NUS Medical School, 8 College Road, 169857, Singapore.

^4^Department of Infectious Diseases, Singapore General Hospital, 20 College Road, 169856, Singapore.

^5^National Neuroscience Institute, 11 Jalan Tan Tock Seng, 308433, Singapore.

^6^Division of Biostatistics and Bioinformatics, Department of Oncology, The Sidney Kimmel Comprehensive Cancer Center at Johns Hopkins, The Johns Hopkins University School of Medicine, Baltimore, MD, USA.

^7^Barbara Davis Center for Diabetes, University of Colorado Denver, Aurora, Colorado, USA.

^8^ImmunoTechnology Section, Vaccine Research Center, NIAID, NIH, Bethesda, MD, USA

^9^Division of Rheumatology, Department of Medicine, The Johns Hopkins University School of Medicine, Baltimore, MD, USA.

*These authors contributed equally to this work.

^†^Corresponding author. Email: [hlarman1@jhmi.edu](mailto:hlarman1@jhmi.edu) | Phone: 410-614-6525


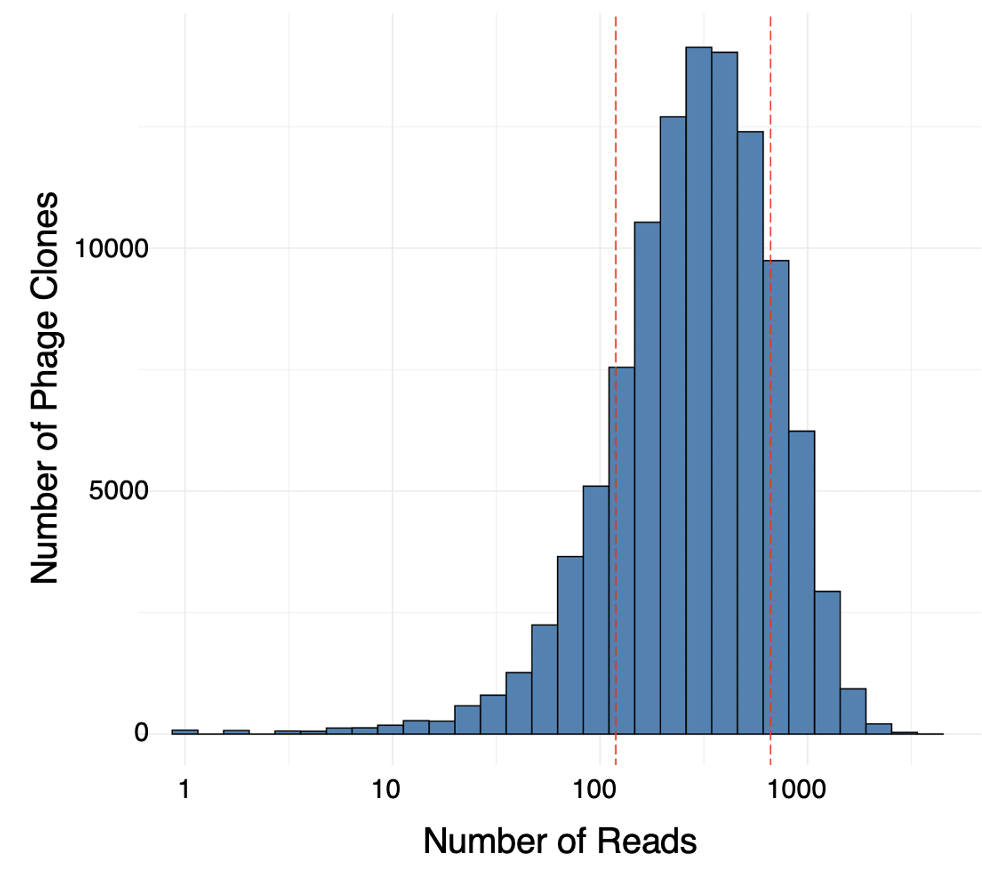


**Figure S1.** Representation of the VirScan phage library: 99.7% of all library members are detected; 68.2% of the library members are within one log of abundance.


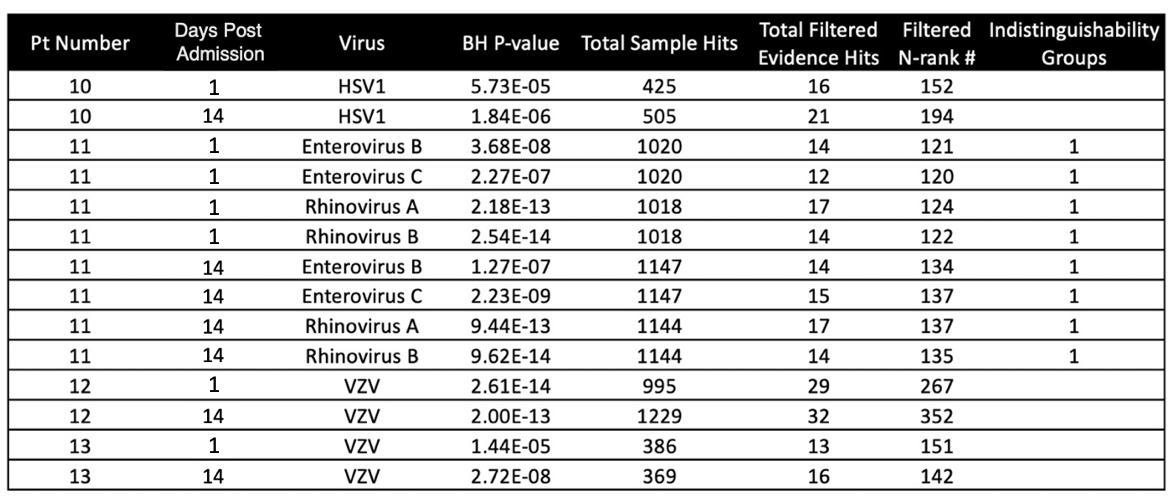


**Table S1.** Single Timepoint AVARDA Analyses: The ‘Day’ column indicates whether a detected viral reactivity was from the initial hospitalization (Day 1) or 14 days later (Day 14). The ‘Virus’ column indicates the viral infection identified by AVARDA. The ‘BH p-value’ column represents the multiple test-corrected AVARDA p-values. ‘Total Sample Hits’ refers to the total number of peptides found to be reactive at a given timepoint. ‘Total Filtered Evidence Hits’ indicates the number of reactive peptides associated with the virus after AVARDA analyses. ‘Filtered N-rank #’ indicates the total number of reactive peptides considered by AVARDA during analysis of the specified infection. The ‘Indistinguishability’ column indicates viruses between which AVARDA was not able to distinguish. Only data from Pts 10-13 are shown and only for viruses that were discordant with nucleic acid testing and the pairwise AVARDA results shown in Table 1.
